# The Nonlinear Impact of Mobile Human Activities on Vegetation Change in the Guangdong–Hong Kong–Macao Greater Bay Area

**DOI:** 10.3390/ijerph20031874

**Published:** 2023-01-19

**Authors:** Qionghuan Liu, Renzhong Guo, Zhengdong Huang, Biao He, Xiaoming Li

**Affiliations:** 1 Research Institute for Smart Cities, School of Architecture and Urban Planning, Shenzhen University, Shenzhen 518060, China; 2 MNR Technology Innovation Center of Territorial & Spatial Big Data, MNR Key Laboratory for Geo-Environmental Monitoring of Great Bay Area, Guangdong Key Laboratory of Urban Informatics, Shenzhen 518060, China

**Keywords:** vegetation change, mobile human activities, nonlinear relationship, land use change, climate change

## Abstract

Vegetation is essential for ecosystem function and sustainable urban development. In the context of urbanization, the Guangdong–Hong Kong–Macao Greater Bay Area (GBA), as the typical urban-dominated region, has experienced a remarkable increase in social and economic activities. Their impact on vegetation is of great significance but unclear, as interannual flow data and linear methods have limitations. Therefore, in this study, we used human and vehicle flow data to build and simulate the indices of mobile human activity. In addition, we used partial least squares regression (PLSR), random forest (RF), and geographical detector (GD) models to analyze the impact of mobile human activities on vegetation change. The results showed that indices of mobile human and vehicle flow increased by 1.43 and 7.68 times from 2000 to 2019 in the GBA, respectively. Simultaneously, vegetation increased by approximately 64%, whereas vegetation decreased mainly in the urban areas of the GBA. Vegetation change had no significant linear correlation with mobile human activities, exhibiting a regression coefficient below 0.1 and a weight of coefficients of PLSR less than 40 between vegetation change and all the factors of human activities. However, a more significant nonlinear relationship between vegetation change and driving factors were obtained. In the RF regression model, vegetation decrease was significantly affected by mobile human activity of vehicle flow, with an importance score of 108.11. From the GD method, vegetation decrease was found to mainly interact with indices of mobile human and vehicle inflow, and the highest interaction force was 0.82. These results may support the attainment of sustainable social–ecological systems and global environmental change.

## 1. Introduction

Human–nature relationship is the core scientific issue in earth science research. Vegetation is the most basic unit of our earth, and the study of the impact of human activities on vegetation change is a hot topic. Vegetation can reduce chronic diseases and premature death and contribute to the well-being of city dwellers [1]. In addition, vegetation is an essential element for sustainable development in urban agglomerations [2,3] as it consolidates ecological security and contributes to improvements in society’s physical and mental health [4]. Therefore, healthy vegetation is important to the sustainable development of urban areas [5,6]. However, previous research has shown that increasing intensities in human activity further fragment vegetation in urbanizing regions, which significantly influences the structure and function of vegetation ecosystems, resulting in a series of environmental problems, including the urban heat island effect and biodiversity loss [7]. In particular, over the past few decades, the urban agglomeration has led to global urban population growth and sprawl [8,9]. Thus, more researchers have focused on the green environments of cities. Especially research on the driving mechanisms of vegetation change has always been of great interest. In the context of global climate change, previous studies have revealed that temperature and precipitation affect vegetation growth and vegetation phenological changes [10]. However, quantizing the impact of human activities on vegetation change has always been the focus topic because of the inconsistency of human activity data.

Research has shown that anthropogenic factors have more varied impacts on vegetation change, including positive impact [11] and negative impact [12]. For example, studies have reported that carbon dioxide emitted from human activities is the main factor driving the increase in vegetation cover [13,14]. Afforestation is another vital factor in improving vegetation greening in China [15]. Furthermore, population and gross domestic product (GDP) were other human activities that caused the vegetation change [11]. In addition, comprehensive human activities of the human footprint, anthromes, global human modification, and low-impact areas were four maps used for the analysis of the relationship between synthetical human activities and vegetation. Among them, the human footprint was mapped based on the population, land transformation, and road accessibility [16]. Anthromes were made with population, land use, and vegetation [17]. Global human modification used more than 13 factors to map the indicator of human activity [18]. In addition, low impact areas were from the classified land use data and excluded the area of urban, cropland, artificial forest land, and night-time lights [19]. In addition, the residual analysis model was used to extract the indirect anthropogenic factors [20]. However, the above-mentioned traditional indices of human activity were made by remote data and statistical data and are a form of static human activities. Because of frequent and intensive human activities in cities, the flow and interaction between humans increased. The notable feature of mobile human activities was the complexity of the mobile, network, and interactive in an urban area [21]. Vegetation is undoubtedly affected by mobile human activity. In addition to the statistical population, the human activities of human travel flow, as well as traffic flow that cannot be monitored by conventional remote or statistical methods exist. Considering the population flow relationships could project real human activities [22]. Human trajectories and hotspots were more widely recognized in the wide world city [23]. However, mobile human activity has never been considered. How quantifying the relationship between mobile human activities and vegetation change was an urgent necessity. Therefore, in the context of mobile human activities, how to reflect the feature of mobile human activities is a significant question but is still lacking in current research. Neglecting the mobile human activities will cause imperfections in understanding the mechanisms driving vegetation change.

The Moderate Resolution Imaging Spectroradiometer (MODIS) vegetation index of Normalized Difference Vegetation Index (NDVI) and Enhanced Vegetation Index (EVI) are commonly used to reflect vegetation cover and ecological quality. The vegetation index is usually affected by many factors, such as clouds, shadows, climate, and environment, and the relationship is more complicated [24]. However, researchers are more inclined to quantify the correlation between vegetation change and driving factors based on linear regression methods, including correlation analysis, multiple regression methods, and geographically weighted regression methods [25,26]. Spatial statistical models consider spatial effects through correlation and regression modeling [27]. This concept can be used to quantify the impact of driving factors on vegetation change. Research showed that geographically weighted regression models could quantify driving mechanisms by calculating the relationships between regression-independent and dependent variables [28]. However, the interactions between climate change, anthropogenic activities, and vegetation are complex under changing global conditions [29]. However, the relationship between vegetation change, climate change, and human activities are coupled [30]. Common regression methods based on the assumption of linearity between independent and dependent variables have great limitations. Random forest (RF) [31] and geographical detector (GD) [32] models are two typical complex regression methods. RF is quantizing single-factor contribution, and the GD module supports the interaction detection of two-factor, which provides an important criterion for solving complex relationship problems. Therefore, some studies have quantified driving mechanisms of vegetation change or ecosystem stability using RF and GD [33,34]. These results showed that the RF method could well diagnose the real relationship between rainfall, temperature, and vegetation in different regions. However, most of the studies obtained results based on a small number of samples, such as administrative boundaries. On the contrary, machine learning methods require a large amount of sample data. Therefore, the contradiction will cause accidental errors.

Overall, this research aimed to answer the following questions: how to build the indices of mobile human activity? And how to quantify the impact of mobile human activities on vegetation change in the Guangdong–Hong Kong–Macao Greater Bay Area (GBA)? In this study, we built the indices of mobile human activity based on the human travel flow and vehicle flow data and used the normalized difference vegetation index (NDVI) and enhanced vegetation index (EVI) to detect vegetation changes and recognize the driving forces of vegetation change by comparing the discrepancy between the linear method of partial least squares regression (PLSR) and nonlinear methods of RF and GD.

## 2. Materials and Methods

### 2.1. Study Area

**Figure 1:**
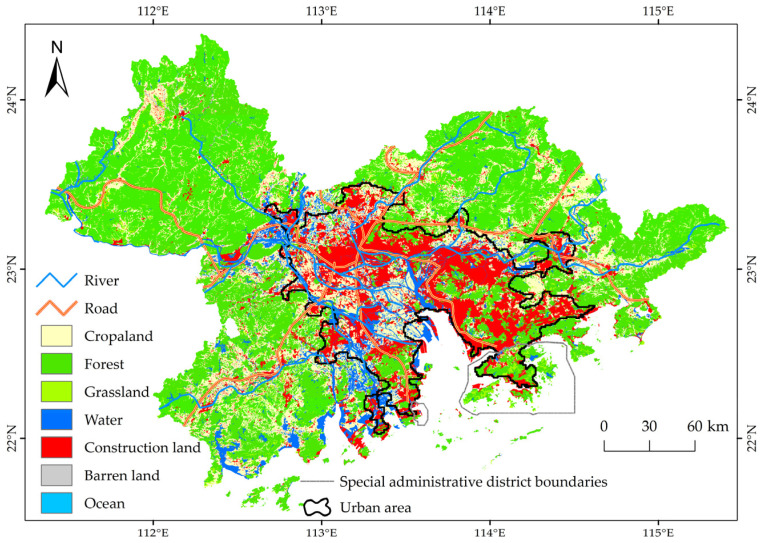
Study area in the Guangdong–Hong Kong–Macao Greater Bay Area. The land cover data were taken from [35].

The GBA (111–116° E, 21–25° N) is a typical transboundary urban agglomeration in China and consists of 11 cities covering a total area of 56,611 km². The region is urbanized with a high level of integration (Figure 1). The proportion of the vegetated area (including cropland, forest, and grassland) accounts for approximately 77.50% of the total land area [35]. The GBA has been experiencing substantial growth in the population and economy. Mobile human and vehicle flow have become prominent features of the GBA, and rapid urbanization and high-intensity land development have generated considerable pressure on vegetation, causing environmental problems [36]. Therefore, identifying the driving mechanism of vegetation change in the new social environment has become a valuable and fundamental topic of research for regional ecosystem governance and sustainable development in the GBA.

In addition, to explore vegetation changes in urban areas, we identified the urban area zone based on the threshold of night light data (greater than 4000) from 2000 to 2019, which accounted for one-quarter of the GBA (Figure 1).

### 2.2. Data and Processing

#### 2.2.1. Vegetation Index

MODIS vegetation index of time-series NDVI and EVI products (MOD13Q1 Version 6, National Aeronautics and Space Administration, SD, USA) data were collected from 2000 to 2019 with 16-day composite products at 250 m resolution. We obtained data from the NASA Land Processes Distributed Active Archive Centre (LPDAAC). Because vegetation in the GBA is dominated by evergreen vegetation, the annual period was selected for January to December as the vegetation growing season, referring to research on vegetation phenology [37]. We used the mean synthesis method to process the annual NDVI and EVI images, and the data were projected to WGS84/Albers Equal Area Conic. Additionally, to reduce disturbances caused by cloud, shade, and atmospheric radiation, NDVI and EVI data were smoothed using the Savitzky–Golay filter in the TIMESAT tool in MATLAB [38].

Although NDVI is the most commonly used vegetation index to signify vegetation, previous studies have shown that EVI data have better effects in areas with higher vegetation coverage [39]. Because of the visible difference in vegetation coverage between urban (low vegetation cover) and rural (high vegetation cover) areas in the GBA, both NDVI and EVI data were used to denote vegetation in this study.

#### 2.2.2. Flow Data

Car navigation data were purchased from the AutoNavi Maps Future Transportation Research Center (Guangzhou, China), including all vehicle navigation directions for the start and end points within the GBA during 2019. CONCOR clustering was used to converge the points scattered throughout the GBA into points based on county-level administrative units, and a statistical frequency method based on the navigation data was conducted to acquire inflow and outflow information of vehicle flow at a resolution of 250 m × 250 m.

Mobile phone signaling data were obtained from China Unicom’s Smart Footprint. The data were obtained for 2018/2019 onwards and processed into inflow and outflow data of the population at a resolution of 250 m × 250 m using the method of Tu et al. (2020) [40].

#### 2.2.3. Other Factors Collection and Processing

Population count data were obtained from WorldPop from 2000 to 2019 at a spatial resolution of 100 m. Nighttime light data were collected from 2000 to 2019 at a spatial resolution of 1000 m [41], and fine particulate matter (PM_2.5_) data at 1000 m resolution were collected from 2000 to 2019 [42]. Air temperature, precipitation, and radiation data were collected from 2000 to 2018 at a spatial resolution of 0.1° [43]. To prepare for the driver analysis, the trend change for all time-series data was calculated using the Theil–Sen analysis methods. The details are presented in Figure S1 (Supplementary Materials). To ensure that the datasets were consistent and comparable with NDVI and EVI, all driving factors were projected onto WGS84/Albers Equal Area Conic projections at the same spatial resolution of 250 m.

The details of the processing are listed in Table 1.

### 2.3. Methods

**Figure 2:**
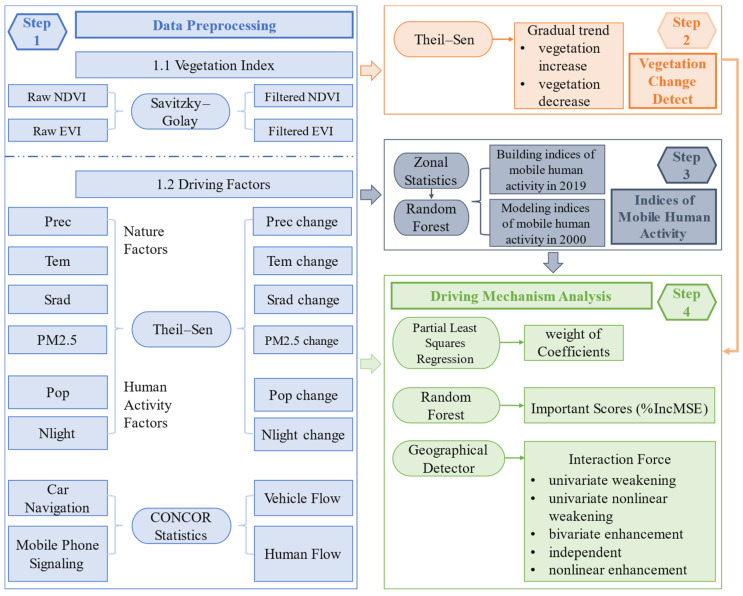
The framework between vegetation change and its driving factors.

The framework of the vegetation change and its driving factors includes four parts: data preprocessing, vegetation change detection, indices of mobile human activity construction, and driving mechanism analysis of vegetation change (Figure 2). The data preprocessing includes obtaining the vegetation index with the influence of clouds and noise removed, which was processed using Savitzky–Golay filtering, acquiring factor change data using a Theil–Sen trend analysis (e.g., Prec, Temp, and Srad, etc.), and obtain flow frequency data through CONCOR method for car navigation and mobile phone signaling data. The second part of vegetation change detection is disposed of in the S-G filtered vegetation index by Theil–Sen trend analysis. The third part of building and modeling the indices of mobile human activity in 2019 and 2000, respectively. The fourth part of the driving mechanism analysis of vegetation change used PLSR analysis, RF regression analysis, and the GD factor interaction detection method. The following subsections provide further details on each method.

#### 2.3.1. Construction of the Indices of Mobile Human Activity

How to reflect the indices of mobile human activity and fusing them with the vegetation index is one of the key issues to be solved. In the study, the construction of indices of mobile human activity was divided into two steps: first, convert flow data expressed as latitude and longitude into the data represented by a grid, and then simulate mobile human activity indices based on correlation factors and regression model.

**Figure 3:**
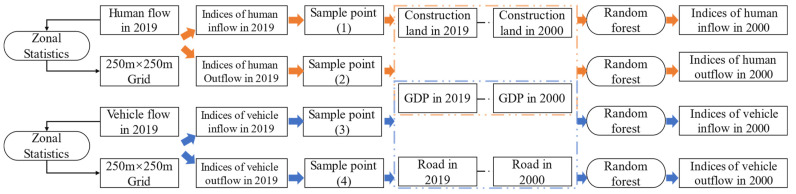
The framework of building the indices of mobile human activities.

We first converted the vehicle flow data in 2019 to the grid of 250 m × 250 m. Then, we statistic the human inflow and human outflow, respectively. Because vehicle flow was related to GDP and road networks [44], therefore, we used the GDP and road networks in 2000 as a regressor (Table 2), using RF regression to fit the relationship between human inflow and outflow with the regressor, respectively. The accuracy of the fitted model was verified by statistical parameters (Table S1, Supplementary Materials). Based on the fitted model, we then simulated the vehicle inflow and vehicle outflow in 2000, respectively. The details of the processing are presented in Figure 3.

Human travel flows were affected by GDP and land use [45]. Therefore, the inflow and outflow information of human travel flow in 2000 was modeled by RF methods combining GDP and construction land rate factors. The details of the processing were similar to the indices of vehicle inflow and outflow. For a detailed description of the RF model, please see Section 2.3.2. The processing procedure was performed in the ‘randomForest’ package of the R language.

#### 2.3.2. Change Detection Methods

The Theil–Sen trend analysis is a nonparametric estimator method. This method is insensitive to measurement errors, robust to outliers [46], and suitable for the trend analysis of long-term series data. Therefore, we used it to analyze vegetation changes in our research. The calculation is expressed as Formula (1):

(1) $$\beta =Median\left(\frac{y_{j}-y_{i}}{j-i}\right)\;\forall j>i$$

where Median is the median value of the slope of the trend, and $y_{i}$ and $y_{j}$ are the time-series values for adjacent years i and j, respectively. β>0 indicates that the vegetation is increasing, and β<0 indicates that the vegetation is decreasing.

The Mann–Kendall test [47,48] is a nonparametric method that does not require measured values to be normally distributed and is insensitive to outliers. It is suitable for the trend significance test of long-term series data and is commonly used in significance test methods. The statistic S and standardized value Z were computed as Formulas (2) and (3), respectively:

(2) $$S=\sum_{i=1}^{n-1}\sum_{j=i+1}^{n}sign\left(y_{i}-y_{j}\right)$$

(3) $$Z=\{\begin{array}{c} \frac{s-1}{\sqrt{Var\left(S\right)}}\;for\;S>0\\ 0\\ \frac{s+1}{\sqrt{Var\left(S\right)}}\;for\;S<0 \end{array}\;for\;S=0$$

where S is the statistic of the size of all paired values in a time series of $y_{i}$, $y_{j}$, …$y_{n}$, 2000 ≤ n ≤ 2019, and Var(S) is the standard deviation of S. The significance level of the trend change of the vegetation index was tested by comparing the value of the statistic with the standard normal variate at a significance level of α (0.05). If |Z| ≥ $Z_{1-\alpha /2}$, then the change in the vegetation is declared to be significant.

#### 2.3.3. Attribution Analysis

(1) Partial Least Squares Regression Model

PLSR was a statistical method of finding a linear regression model by projecting predictor and observed variables into a new space. It was first developed by Wold and Albano in 1983 [49]. PLSR was used to find the underlying relationship of two matrices (X and Y). The PLSR model tried to find the multidimensional directions in the X space to explain the multidimensional directions with the greatest variance in the Y space.

PLSR was particularly useful when the prediction matrix had more variables than observations and when there was multicollinearity in the values of X matrice. Therefore, in the study, we used the PLSR method to analyze the driving mechanism of vegetation change. In the study, vegetation change was divided into an increasing pattern and a decreasing pattern. Furthermore, the discrepancy between the rural area and the urban area was obvious in the GBA. From previous research, the different change pattern of vegetation was affected by varieties of driving factors. Therefore, to consider the spatial heterogeneity, we built the model based on the vegetation change pattern to analyze the impact factors of vegetation change in the GBA.

(2) Random Forest

RF model used multiple regression trees to continuously generate training samples and test samples by the bootstrap method (Figure S2, Supplementary Materials). The samples and features were randomly generated. The regression results depend on the vote scores of each regression tree [31,50]. The combination of different driving factors determines the accuracy of the RF regression results. Therefore, the RF method is a nonlinear process that determines the contributions of regression factors.

The RF regression method is a widely recognized and accepted method in the study of land use change in China [51,52,53] and global areas [54]. Therefore, we used this method to test the correlation between vegetation change and the driving factors. In the study, we collected 10 driving factors as a regressor (Table 1). The increase in the mean square error (%IncMSE) values generated during the RF regression model means how much model accuracy would reduce when one factor was eliminated. It can be expressed as the contribution of the driving factors to the model [34]. In the study, it is used as the contribution of the driving factors to vegetation change.

RF can handle high-dimensional data and multicollinearity and is insensitive to overfitting. However, it is sensitive to the sampling design scheme [55]. In this study, a total of 785,829 valid sample points were taken in the GBA. We divided the points into a training sample (550,080, 70%) and a testing sample (235,749, 30%) for the regression of the RF model. We implemented several pre-run tests to evaluate the sensitivity and robustness of the model (Table S2, Supplementary Materials). The relationship between each factor and the vegetation index is shown in Figure S3 (Supplementary Materials). After the test, we decided to use 4 predictor variables (mtry) for each tree split and 500 trees (ntree) in the model. In addition, we assessed the accuracy of the RF regression model using the parameter of root mean square error (RMSE) and coefficient of determination (R²). The better the model performance, the higher R², and the lower RMSE.

(3) Interaction Detection Methods

The GD method can detect the spatial relationship between land surface change and driving factors and the interactions of driving factors with land surface change. This method is more suitable than a linear model for explaining the nonlinear relationship between vegetation change and driving mechanisms [32], and its robustness was verified [56]. Vegetation change is a typical surface change process; thus, we assumed that GD could detect the driving mechanisms of vegetation change reasonably well. The GD comprises four modules: factors, interactions, risks, and ecology. An interaction detector was used in this study.

The interaction detector can identify the interactions of bivariate factors and indicate whether the interactions between factors $X_{1}$ and $X_{2}$ are weakened, enhanced, or independent of the influencing variable Y [32]. The degree of impact is denoted by the q value, which was calculated using the Formulas (4) and (5):

(4) $$q=1-\frac{\sum_{h=1}^{L}N_{h}\sigma_{h}^{2}}{N\sigma^{2}}=1-\frac{SSW}{SST}$$

(5) $$SSW=\sum_{h=1}^{L}N_{h}\sigma_{h}^{2},\;SST=N\sigma^{2}$$

where h=1,2, …,L is the strata of variable Y (in the study, it represents vegetation index or factor X (in the study, it represents human inflow, human outflow, vehicle inflow, vehicle outflow, Prec, Temp, etc.); N is the numbers of sample units in the entire region; $N_{h}$ is the number of sample units in the subregion h; $\sigma^{2}$ is the global variances Y over the entire study region; $\sigma_{h}^{2}$ is the local variances in the subregion h; and SSW and SST are the sum of squares and the total sum of squares, respectively. The q value detects the extent to which factor X explains variable Y. The range q of the values is between 0 and 1, where the higher the q value, the stronger the impact of driving factor X on Y.

The interactive relationship can be divided into five categories by comparing the interactive q value of two arbitrary factors ($q\left(x_{1}\cap x_{2}\right)$) and the q value of two factors $q\left(x_{1}\right)$, $q\left(x_{2}\right)$, respectively [57]. The relationships shown were univariate weakening, univariate nonlinear weakening, bivariate enhancement, and independent and nonlinear enhancements. The details are presented in Table S3 (Supplementary Materials).

## 3. Results

This study was focused on the key issue of the impact of mobile human activities on vegetation changes. We built the indices of mobile human activity, followed by a trend analysis of vegetation changes. We then quantified the impact of mobile human activities on vegetation change. The main result showed that the nonlinear interaction of HFlowIn and TFlowIn was found to enhance the trend of vegetation decrease, with a maximum interaction force of 0.82. The details result is presented in Section 3.1, Section 3.2, Section 3.3 and Section 3.4.

### 3.1. Changes in Mobile Human Activity

**Figure 4:**
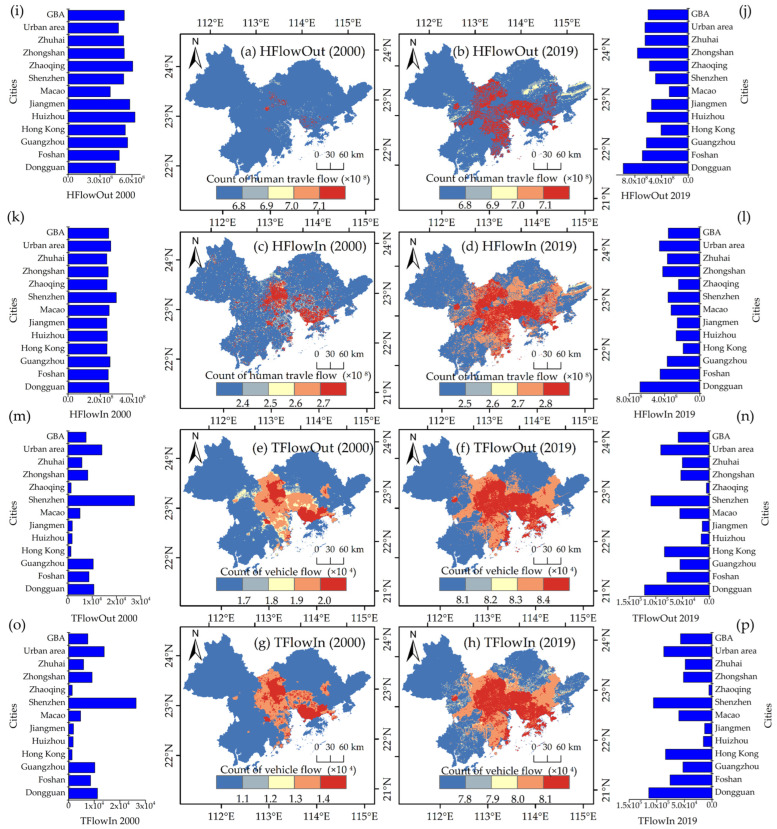
Spatial change trend for the indices of mobile human activity from 2000 to 2019. (**a**,**b**) depicted the indices of human travel outflow in 2000 and 2019, respectively. (**c**,**d**) depicted the indices of human travel inflow in 2000 and 2019, respectively. (**e**,**f**) depicted the indices of vehicle outflow in 2000 and 2019, respectively. (**g**,**h**) depicted the indices of vehicle inflow in 2000 and 2019, respectively. The bar graph from (**i**–**p**) on the left and right were the statistical result of the indices of mobile human activity.

Figure 4 shows that human and vehicle flows increased from 2000 to 2019, with an average increase of 1.43 and 7.68 times, respectively. The human flow in 2019 was approximately 1.1 times higher than that in 2000. In the past 20 years, the inflow and outflow of humans in the urban area of the GBA have increased 1.73 and 1.37 times, respectively. Among the various cities, Dongguan is the city with the most obvious increase in human flow, whose inflow and outflow increased 2.67 and 2.19 times, respectively. The vehicle flow in 2019 was significantly higher than that in 2000, and its vehicle inflow and outflow in 2019 were higher by 7.59 and 7.77 times than in 2000, respectively.

The urban area was higher than the rural area, and the vehicle inflow and vehicle outflow were approximately 87,243 and 90,819, respectively. Shenzhen’s vehicle inflow and vehicle outflow were significantly higher than those in other cities, at approximately 106,006 and 109,185, respectively. Dongguan and Hong Kong had higher vehicle inflows of approximately 114,540 and 84,155, respectively.

### 3.2. Trend Changes in Vegetation

**Figure 5:**
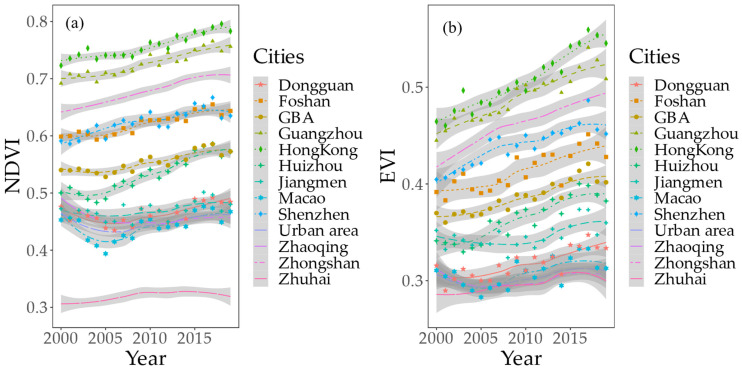
Overall change trend for vegetation from 2000 to 2019. (**a**) NDVI change trend (**b**) EVI change trend.

From 2000 to 2019, the vegetation index in the GBA showed a steadily increasing trend. The annual average increases in the NDVI and EVI were 0.0024 (*p* < 0.05) and 0.0023 (*p* < 0.01), respectively. In general, Hong Kong, Shenzhen, Guangzhou, and Foshan were the five cities with relatively significant increases in the vegetation index, showing values higher than the annual average level in the GBA. NDVI data showed that Huizhou (0.0053, *p* < 0.01) and Shenzhen (0.0038, *p* < 0.01) had the fastest vegetation growth, whereas EVI data showed that Hong Kong (0.0045, *p* < 0.01) and Huizhou (0.0035, *p* < 0.01) had the fastest vegetation growth (Figure 5).

**Figure 6:**
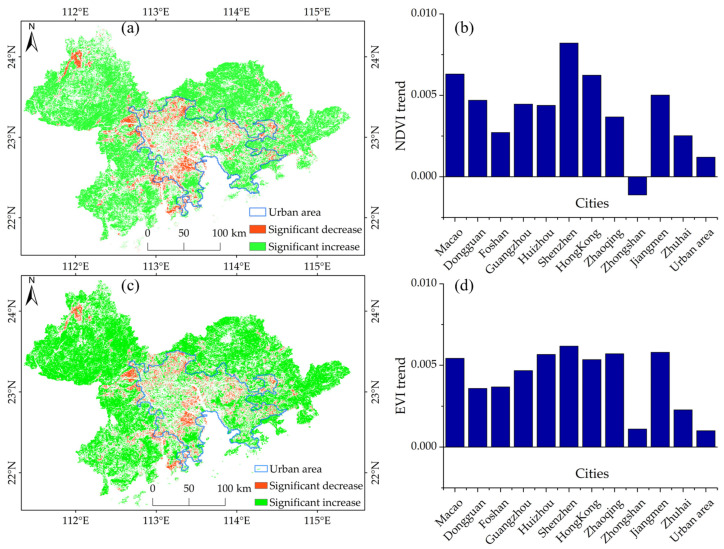
Spatial change trend for vegetation from 2000 to 2019. (**a**,**b**) denoted the gradual change based on NDVI; (**c**,**d**) denoted the gradual change type based on EVI.

From 2000 to 2019, NDVI and EVI data in the GBA showed significant trends, increasing by approximately 64.92% and 66.98%, respectively. Areas with significant vegetation reductions were concentrated in the central part of the GBA. From the perspective of different cities, the growth rate of the vegetation index in Shenzhen was the fastest, with increases in the rates of NDVI and EVI being approximately 0.008/a and 0.006/a, respectively. Notably, during the same period in Zhongshan, the NDVI showed a downward trend, with a decreasing rate of −0.002/a (Figure 6). In contrast, the EVI data showed an increasing trend for all 11 cities.

### 3.3. Importance Factors of Driving Vegetation Change

#### 3.3.1. The Nonlinearly Impact of Driving Factors on Vegetation Change

**Figure 7:**
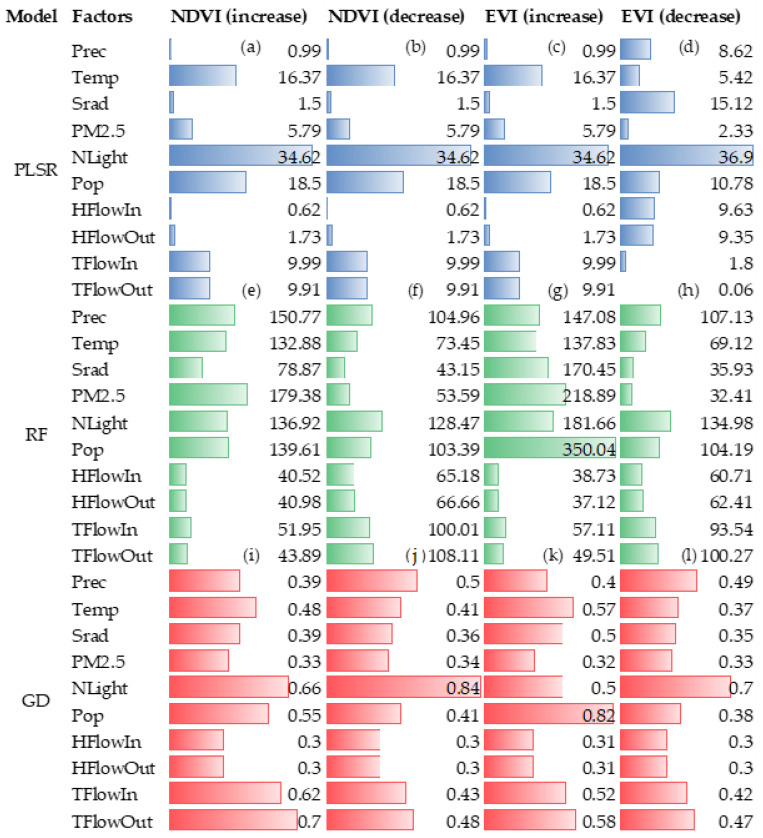
The importance score of driving factors to vegetation change based on partial least squares regression, random forest, and geographical detector methods. (**a**,**e**,**i**) were the important score of impact factors in NDVI increase change based on partial least squares regression, random forest, and geographical detector, respectively. (**b**,**f**,**j**) were the important score of impact factors in NDVI decrease change based on partial least squares regression, random forest, and geographical detector, respectively. (**c**,**g**,**k**) were the important score of impact factors in EVI increase change based on partial least squares regression, random forest, and geographical detector, respectively. (**d**,**h**,**l**) were the important score of impact factors in EVI decrease change based on partial least squares regression, random forest, and geographical detector, respectively.

As shown in Figure 7, based on the NDVI and EVI vegetation indices, the linear regression between vegetation change and climate or human activity factors was a weak relationship, and the regression R² was below 0.1 (Table S4, Supplementary Materials). Specifically, based on the NDVI vegetation index, the increase in vegetation had a weak correlation (with the weight of coefficients in the range of 10 to 30) with the vehicle flow (TFlowIn and TFlowOut) and human flow (HFlowIn and HFlowOut) (Figure 7a,c); and vegetation increase showed no significant correlation with climate factors of Prec, Temp, and Srad (with a weight of coefficients in the range of 0 to 20). Based on the EVI, the vegetation increase had no significant relationship with human activity and climate change. In addition, both NDVI and EVI also showed that vegetation decrease had no significant relationship with any of the driving factors (with the weight of coefficients below 10) (Figure 7c,d).

Figure 7 shows that the importance score of climatic factors in relation to vegetation change is high, whereas the contribution of human activity factors is relatively low according to the %IncMSE values of the RF regression models. Based on both the NDVI and EVI, the human activity factors of NLight were shown to have a significant impact on vegetation decrease, with %IncMSE values of 139.81 (Figure 7f,h). In addition, the impact of human and vehicle flow on vegetation decrease was shown to be significant, with the TFlowIn with %IncMSE values of 108.11. For vegetation increase, it was shown to be greatly affected by climatic factors of PM_2.5_, Prec, and Temp, with the %IncMSE values being 218.89, 166.40, and 145.56, respectively (Figure 7e); Pop was also found to play an important role in the increase in vegetation changes (Figure 7f).

Based on the NDVI and EVI, the Q values showed that human activities had higher values than climate factors. This means that human activities have a more significant impact on vegetation change than climate factors. For both vegetation increase and decrease, the effects of NLight, vehicle flow (TFlowIn and TFlowOut), and Pop were more obvious than other factors, with average Q values of 0.68, 0.56, and 0.54, respectively (Figure 7i–l). In addition, Temp and Prec also have great contributions to vegetation increase change, with Q values 0.57 and 0.40, respectively (Figure 7k).

#### 3.3.2. Interactions of Factors with Vegetation Change

**Figure 8:**
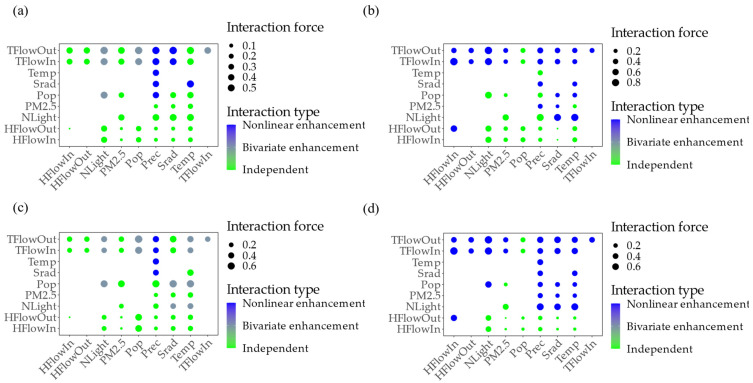
Interactions forces of driving factors to vegetation change. (**a**) NDVI increase; (**b**) NDVI decrease; (**c**) EVI increase; and (**d**) EVI decrease.

Based on the GD methods, the main interactions between factors include bivariate enhancement and independent and nonlinear enhancement in the GBA. Natural factors, such as PM_2.5_ and Temp, tend to have independent influences on vegetation change (green scatter in Figure 8), whereas mobile human activity factors mainly exhibit nonlinear enhancement effects. In addition, the nonlinear interaction of human flow (HFlowIn and HFlowOut) and vehicle flow (TFlowIn and TFlowOut) was found to enhance the trend of vegetation decrease (blue scatter in Figure 8b,d), with a maximum interaction force of 0.82 between HFlowIn and TFlowIn. In contrast, the nonlinear interaction between Prec and other factors enhanced the change in vegetation (blue scatter in Figure 8a,c). The highest interaction force of 0.63 was between Prec and Pop.

### 3.4. Accuracy of Random Forest Regression

**Figure 9:**
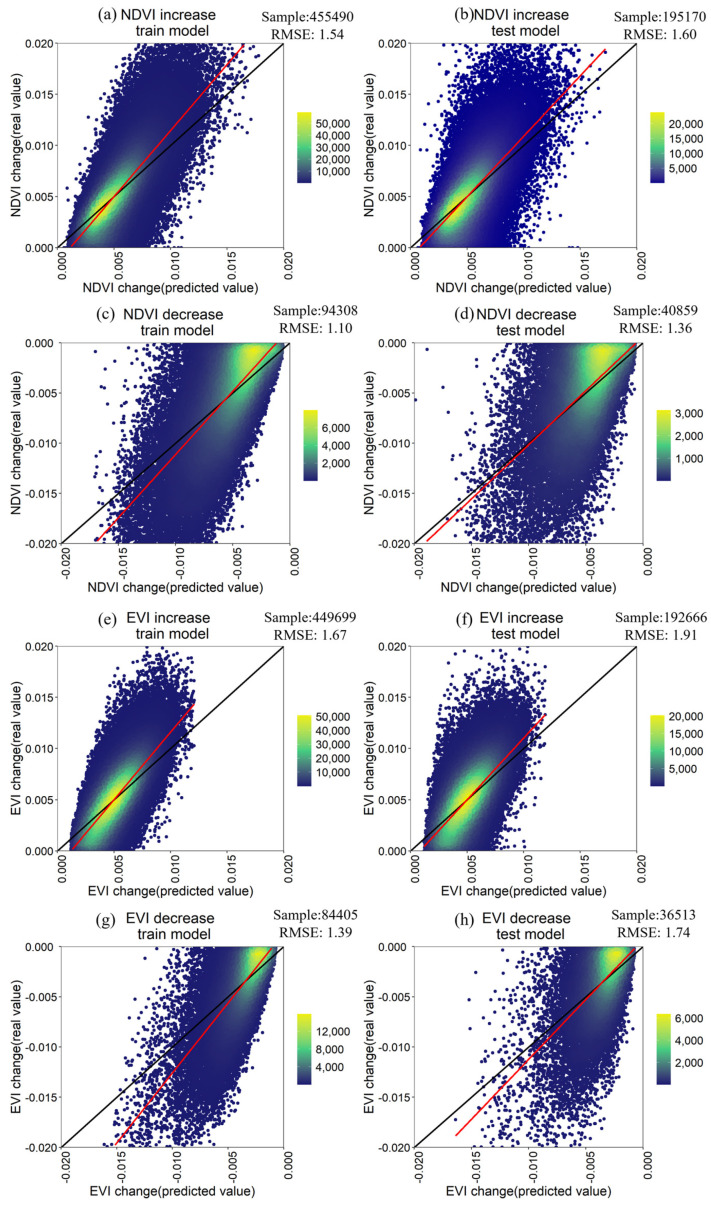
Accuracy assessment of Random Forest regression. (**a**,**b**) the accuracy of random forest in NDVI increases based on the train and test models, respectively. (**c**,**d**) accuracy of rando forest in NDVI decreased based on the train and test model, respectively. (**e**,**f**) accuracy of rando forest in EVI increase based on the train and test model, respectively. (**g**,**h**) accuracy of rando forest in EVI decreased based on the train and test models, respectively.

The sample point was divided into train points and test points to analyze the impact of driving factors of climate and human activities on vegetation change. The RF model performance was analyzed by contrasting the differences between the real and predicted NDVI values in the GBA (Figure 9). In the study, we compared the real and predicted NDVI values in the train and test models, respectively. From the result, the RMSE between the real and predicted NDVI value was below 2. Therefore, the results of the RF regression model were reasonable in quantizing the importance scores of factors for vegetation change.

## 4. Discussion

### 4.1. How the Indices of Mobile Human Activity Replenish the Data of Human Activities

Previous research, based on the population, GDP, and land use intensity, showed that the impact of human activities on vegetation change was ambiguous. On the one hand, in decentralized city areas, the development of traditional agriculture and urban agriculture made a greater contribution to vegetation greening in some parts of Europe, Australia, and North America [58]. Additionally, in urban areas, vegetation increase was usually associated with park expansion and artificial greening [59]. On the other hand, previous studies have concluded that human activities data affected the accuracy of mechanism analysis of vegetation change [60]. Therefore, in the urbanization and flourishing urban areas of GBA, human activities data need to be updated.

In the study, we built and reconstructed the indices of vehicle inflow, vehicle outflow, human inflow, and human outflow (Figure 4) and supplemented and reanalyzed the impact of human activities on vegetation change in GBA. Overall, the results of this study showed that the indices of mobile human activity have a more significant impact on vegetation change than population. We thought there might be two reasons for this. First, direct human activities were stronger than indirect human activities. This was consistent with the research that flow activities were the main form of human characteristics in cities [61]. Therefore, when we tried to detect the impact of human activities on vegetation by a direct factor of mobile human activities, we found that they had a significant impact on vegetation change. Second, human activities are large complex systems and will have a significant effect only when a complete human system is formed, while the intensity of the action of the static factor on vegetation is negligible [62]. However, in the previous research, the mobile human activity factors had been neglected. The latest research showed that the mobile population has increased since the end of the 20th century [63]. The common human activity index underestimated the intensity of human activities. In the GBA, with low vegetation cover, increasing human activities will have a significant impact on the vegetation change. Therefore, through the study, we suggest that mobile human activities are important factors that affect vegetation change.

### 4.2. Utilize Nonlinear Methods to Quantify the Complex Relationship between Vegetation and Impact Factors

Influenced by the complex environment, the relationship between vegetation, climate, and human activities is difficult to quantify. Previous research has attributed the vegetation change to a complex interaction process of multiple factors. However, in the conventional analysis, the linear method was commonly used. Zhao et al. (2018) used the linear regression method to analyze global vegetation activity and its driving factors [26]. Wu et al. (2020) used the partial correlation analysis to quantify the impacts of climate and human activities-induced vegetation variations [64]. The linear method revealed the direct influence of precipitation and temperature on vegetation but downplayed the interactive process between factors and vegetation.

Yang et al. (2021) used the nonlinear method of geographically and temporally weighted regression to evaluate the NDVI responses to climate change [25]. Liu et al. (2021) used RF to explain the cause of the vegetation change [34]. Although these studies have used nonlinear methods to diagnose vegetation change and quantify the reasons for vegetation change, they did not take both single-factor and double-factor interactions into consideration. Especially we found the existence of mobile human activities in the GBA. Mobile human activities and their connected environment usually form a complex relationship dominated by networks [61]. Therefore, in the study, we used both linear and nonlinear methods to identify the contribution of driving factors to vegetation change. Compared with the linear method of PLSR, the RF and GD showed a more significant result in quantizing the relationship between vegetation and driving factors and reflected the impact of traffic flow and climate factors, as well as the interaction of traffic flow and human flow on vegetation change. The result was consistent with the previous research on the nonlinear impact of the built environment on human behavior [65]. This is also consistent with the fact that human and traffic flow usually increase the changes in the intensity of human activities (see Section 4.3) and the fact that the relationship between mobile human activities and vegetation is indirect.

Furthermore, in previous research, the administrative unit and watershed unit was used as the statistic unit, but we used the 250 m grid as the minimum unit, and there was a large amount of data information, which matched the requirements of a large data volume of nonlinear method and reduced the random errors. In addition, different vegetation change trends were affected by different factors. Although previous studies did not consider trends and types in vegetation change [26], we divided the vegetation into greening change and browning change to analyze the important score of impact factor for each change form, which helped to improve the rationality in quantifying the driving mechanism of vegetation change.

### 4.3. Interactions among Anthropogenic Factors Dominated Vegetation Changes in Urban Centers

From 2000 to 2019, the population of the GBA increased by 555.14 million, making it the fastest-growing urban agglomeration in China. Concurrently, the GDP in the GBA increased from 847.13 to 8952.39 billion yuan, an increase of 12.51%. In terms of car traffic, the growth rates of passenger traffic, taxis, and civilian vehicle drivers in Guangdong Province are 3.37 million/year, 2317/year, and 1.87 million/year, respectively.

An increase in population usually leads to the expansion of urban areas and economic development [66], which occupies the vegetation area and reduces the vegetation index. Similar conclusions have been drawn in previous studies. For example, urban expansion may decrease vegetation diversity and increase landscape fragmentation [67,68]. Therefore, the interaction between population and economy may make the trend of vegetation deterioration more significant. Similarly, the increase in vehicle flow due to the increase in the population indicates that the city is vigorous [40] and will increase the construction of infrastructure, such as roads, which may have a negative impact on vegetation.

According to the 2019 China Urban Traffic Report released by Baidu Maps Smart Eye, people in the GBA are more mobile than those in other cities in China. In addition to the high production efficiency and profits brought about by high-intensity work, the operating frequency of travel and traffic has also increased. On the one hand, an increase in the concentration of PM_2.5_ may not be conducive to vegetation growth. On the other hand, the carbon dioxide produced increases photosynthesis in vegetation, which is conducive to vegetation greening [69]. The increase in the urban population in the GBA and the expansion of urban construction land have resulted in the conversion of natural vegetation into artificial vegetation [70]. However, artificial vegetation is more fragile than natural vegetation and more susceptible to human activities [71], thus, exacerbating the trend of vegetation degradation in the urban area of the GBA. Details of the impacts of human activities on vegetation are shown in Figure S4, Supplementary Materials.

### 4.4. Limitations

This study excluded areas with ground vegetation coverage with NDVI and EVI values greater than 0.2. On the one hand, this excluded the inapplicability of the vegetation index in areas with low vegetation coverage; on the other hand, it may have also led to the underestimation of the impacts of human activities or climatic factors on vegetation change. Moreover, other factors related to soil and altitude must be further considered for vegetation changes [72]. Climate also varies across the GBA, and factors such as the impact of typhoons on vegetation need to be considered in further studies. In addition, the indices of human flow and vehicle flow built in the study have no validation data for reference in current research. Although we evaluated the accuracy by model parameter and also connected the indices with the mobile population in China, it will cause uncertainty for the influential intensity of human activities on the vegetation change.

There are limitations to mining spatial relationships among urban objects using statistical and spatial analysis methods based on samples and layers. Owing to the lack of road trajectory data, data for human and vehicle flows were used in the study; the flow data were finally converted to grid inflow and outflow data. Therefore, the relationship between human activity and vegetation was not identified based on the background of the flow space. There were problems owing to the difficulties in converting the raster data into flow data. Notably, relevant preliminary studies have transformed land-use data into flow data [73]. However, further research is required to convert various types of raster data into graph data to construct a flow-space network [74,75]. For the flow space, it would be more favorable to use the deep learning knowledge map to mine the relationship, as this would more efficiently quantify the impacts of human activities on vegetation changes.

## 5. Conclusions

New insights were found in vegetation-driving mechanisms after considering the indices of mobile human activities. The mobile human activity of vehicle flow significantly affected vegetation decrease in GBA. Although vegetation showed a greening trend in the GBA, there was an obvious weakening greenness in the urban area in the GBA. The interaction between humans and vehicle flows reinforces the effect of vegetation browning. Overall, mobile human activities have caused vegetation to decrease in urban areas, but the positive effects of climate factors on vegetation can generally offset the impact of mobile human activities in the GBA. Owing to high-density crowding in the GBA, vegetation is important for improving the health of residents and increasing the production efficiency of enterprises. Thus, it is necessary to use a future perspective to focus on vegetation health in areas of high mobility and agglomeration in the GBA. This could, in turn, meet the economic development requirements and also benefit the urban ecosystem.

## Figures and Tables

**Table 1:** Information about the driving factors.

Type	Factors	Description	Timespan	Spatial Resolution
Human activities factors	HFlowIn	Human inflow change	2018 and 2000	-
	HFlowOut	Human outflow change	2018 and 2000	-
	TFlowIn	Vehicle inflow change	2019 and 2000	-
	TFlowOut	Vehicle outflow change	2019 and 2000	-
	Pop	Population change	2000–2019	100 m
	Nlight	Nightlight change, indicating the economic development of the city	2000–2019	1 km
Climate factors	Prec	Precipitation change	2000–2018	1 km
	Temp	Temperature change	2000–2018	1 km
	Srad	Radiation change	2000–2018	1 km
	PM_2.5_	PM_2.5_ change	2000–2019	1 km

**Table 2:** Information about the factors used for modeling indices of mobile human activity.

Factors	Description	Timespan	Spatial Resolution
GDP	Use for modeling human flow and vehicle flow	2000 and 2019	1 km
Road	Use for modeling vehicle flow	2000 and 2019	-
Construction land	Use for modeling human flow	2000 and 2019	30 m

GDP, gross domestic product.

## Data Availability

The data of MODIS NDVI and EVI were obtained from NASA Land Processes Distributed Active Archive Centre (LPDAAC) (https://lpdaac.usgs.gov/, accessed on 1 September 2022). Driving factors data were obtained from the following source: Population count data were obtained from WorldPop (https://www.worldpop.org/, accessed on 1 September 2022). Nighttime light data were collected from (https://data.tpdc.ac.cn/, accessed on 1 September 2022). Fine particulate matter (PM_2.5_) data were collected from (https://doi.org/10.5281/zenodo.5919481, accessed on 1 September 2022). Air temperature, precipitation, and radiation data were collected from https://doi.org/10.6084/m9.figshare.c.4557599, accessed on 1 September 2022. GDP data was obtained from Resource and Environment Science and Data Center (https://www.resdc.cn/, accessed on 1 September 2022). Construction land was from the Resource and Environment Science and Data Center (https://www.resdc.cn/, accessed on 1 September 2022). Road data in 2000 from the historical map, Planet Map Publishing House, and 2019 from OSM (https://www.openstreetmap.org/, accessed on 1 September 2022). Car navigation data and mobile phone signaling data are available from the author upon reasonable request. The land cover data is from Resource and Environment Science and Data Center (https://www.resdc.cn/, accessed on 1 September 2022).

## Supplementary Materials

The following supporting information can be downloaded at: https://www.mdpi.com/article/10.3390/ijerph20031874/s1, Figure S1: Spatial distribution of driving factors; Figure S2: The processing flow of the Random Forest model; Figure S3 Nonlinear impact of the driving factors on vegetation change; Figure S4 Conception of the impacts of human interactions on vegetation change in central urban areas; Table S1: Model performance parameters for the RF; Table S2: Model performance parameters for the indices of mobile human activity by RF; Table S3: Definition of the interactive relationship; Table S4: Model performance parameters for the driving factors analysis by PLSR.
