# Supplementary material for: The Nonlinear Impact of Mobile Human Activities on Vegetation Change in the Guangdong–Hong Kong–Macao Greater Bay Area
Source: Int J Environ Res Public Health. 2023 Jan 19;20(3):1874. doi: 10.3390/ijerph20031874 (PMC9914965; doi:10.3390/ijerph20031874)
Supplement: Supplementary file 1 [file ijerph-20-01874-s001.zip › ijerph-2022061-supplementary.pdf]

Supplementary Materials

The nonlinear impact of mobile human activities on vegetation change in the Guangdong–Hong Kong–Macao Greater Bay Area

Qionghuan Liu [1, 2], Renzhong Guo [1,2], Zhengdong Huang [1,2], Biao He [1,2], and Xiaoming Li [1,2,*]

1 Research Institute for Smart Cities, School of Architecture and Urban Planning, Shenzhen University, Shenzhen 518060, China

2 MNR Technology Innovation Center of Territorial & Spatial Big Data, MNR Key Laboratory for Geo-Environmental Monitoring of Great Bay Area, Guangdong Key Laboratory of Urban Informatics, Shenzhen 518060, China

Corresponding author. E-mail addresses: lixming@szu.edu.cn.

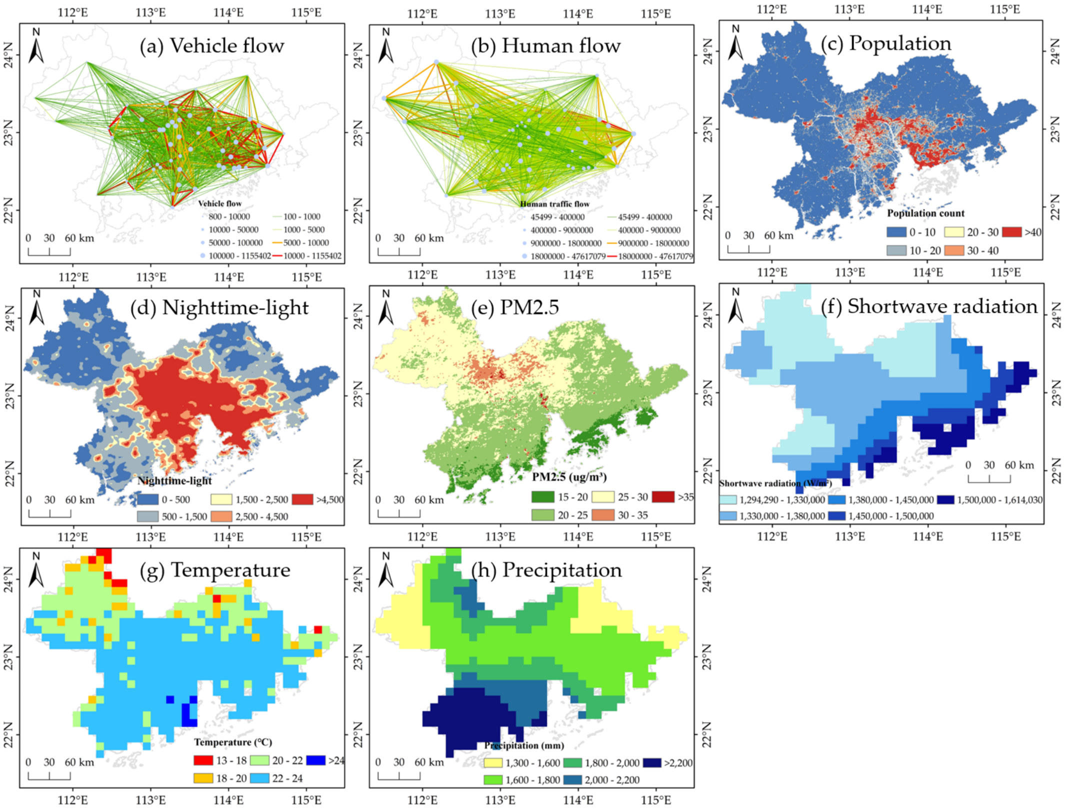

Figure S1. Spatial distribution of driving factors. (**a**) car navigation data (**b**) mobile phone signaling data (**c**) WorldPop data (**d**) nighttime light data (**e**) particulate matter (PM2.5) data (**f**) shortwave radiation data (**g**) temperature data (**h**) precipitation data.

Table S1. Model performance parameters for the driving factors analysis by RF

**Performance parameters**	**NDVI increase**	**NDVI decrease**	**EVI increase**	**EVI decrease**
% Var explained	67.06	64.19	64.88	58.45
R^2	0.66	0.64	0.63	0.57

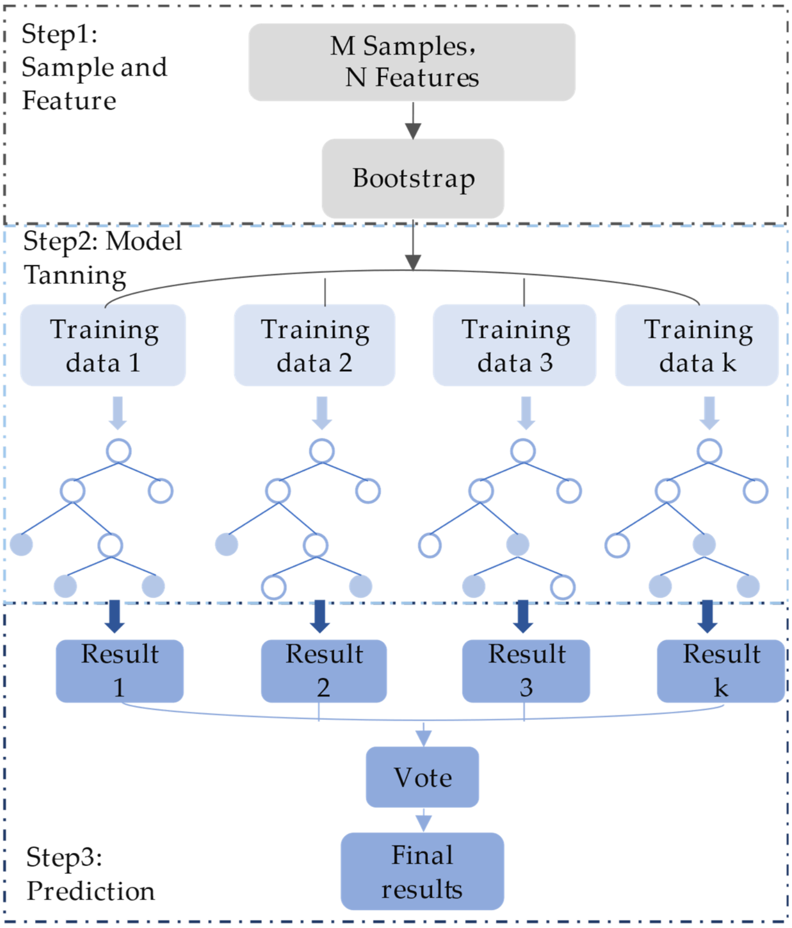

Figure S2. The processing flow of the Random Forest model.

Table S2. Model performance parameters for the indices of mobile human activity by RF

Performance parameters	HFlowOut in 2000	HFlowIn in 2000	TFlowOut in 2000	TFlowIn in 2000
% Var explained	69.01	79.06	82.89	83.41
R^2	0.79	0.80	0.89	0.89

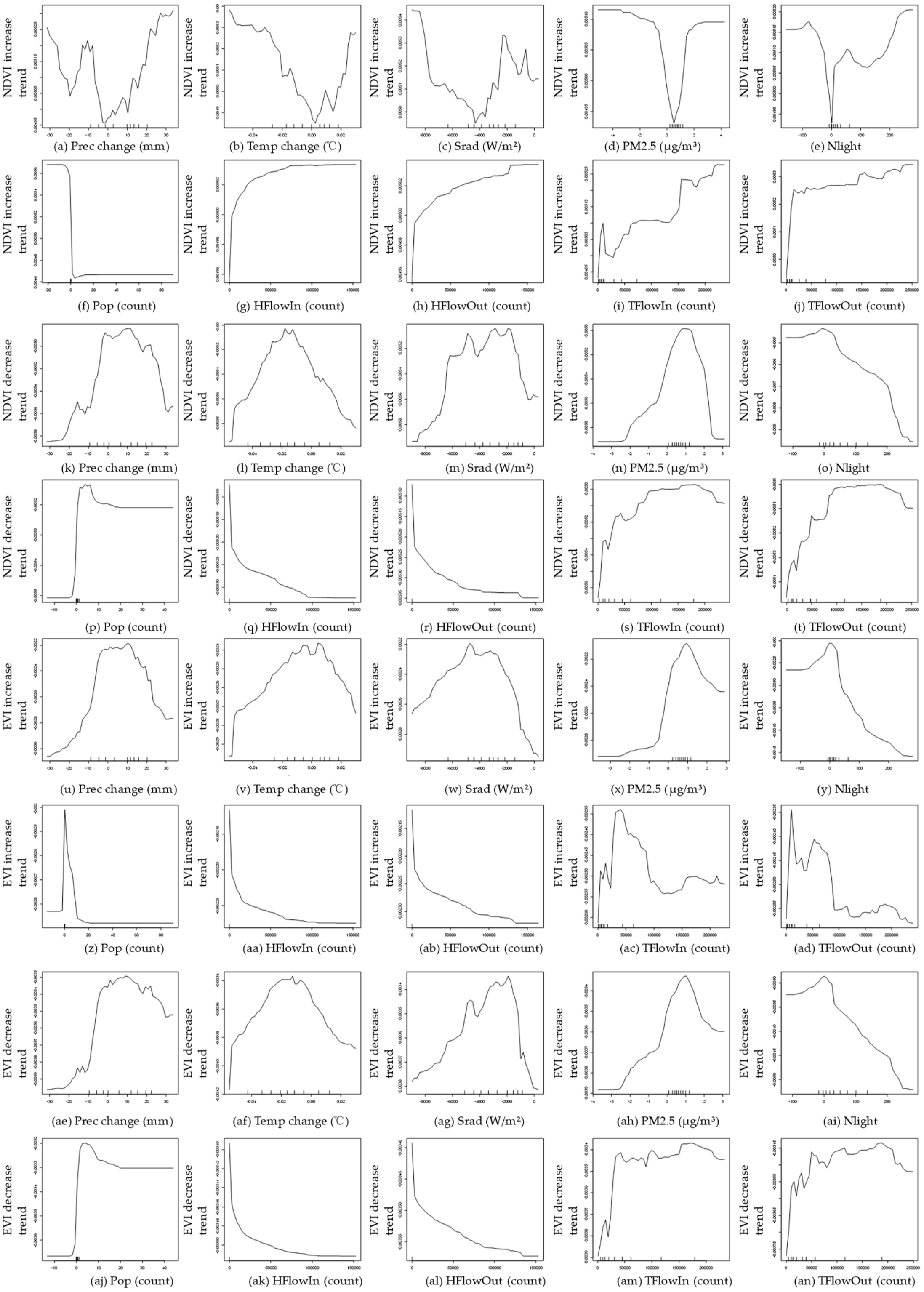

Figure S3. Nonlinear impact of the driving factors on vegetation change

Table S3. Definition of the interactive relationship

Interactive relationship	Definition
Univariate weakening	$q\ ((x_1 \cap x_2)) < \min\ (q(x_1),\ q(x_2))$
Univariate nonlinear weakening	$\min\ (q(x_1),\ q(x_2)) < q(x_1 \cap x_2) < \max\ (q(x_1),\ q(x_2))$
Bivariate enhancement	$\max\ (q(x_1),\ q(x_2)) < q(x_1 \cap x_2) < q(x_1) + q(x_2)$
Independent	$q(x_1 \cap x_2) = q(x_1) + q(x_2)$
Nonlinear enhancement	$q(x_1 \cap x_2) > q(x_1) + q(x_2)$

Table S4. Model performance parameters for the driving factors analysis by PLSR

Performance parameters	NDVI increase	NDVI decrease	EVI increase	EVI decrease
% Var explained	29.66	11.78	28.56	18.86
Adjusted R^2	0.03	0.04	0.02	0.03

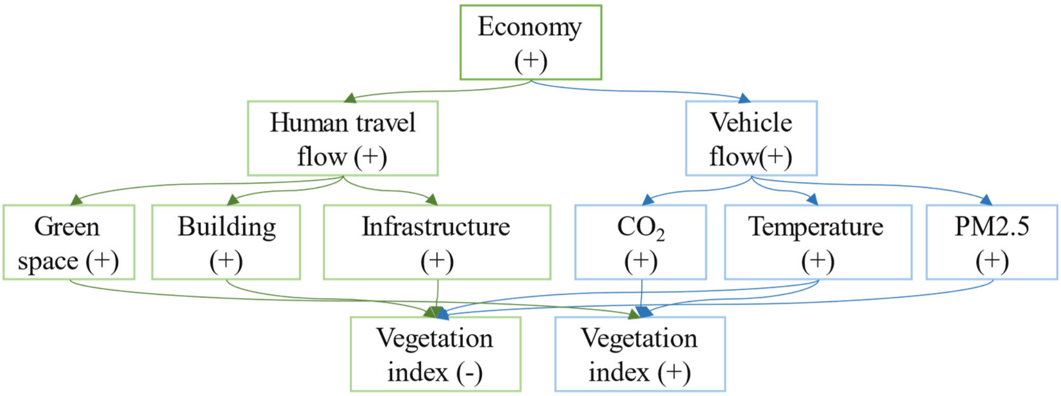

Figure S4. Conception of the impacts of human interactions on vegetation change in central urban areas.
